# Ion-Triggered Hydrogels Self-Assembled from Statistical Copolypeptides

**DOI:** 10.1021/acsmacrolett.1c00774

**Published:** 2022-02-16

**Authors:** Bing Wu, Saltuk B. Hanay, Scott D. Kimmins, Sally-Ann Cryan, Daniel Hermida Merino, Andreas Heise

**Affiliations:** † Department of Chemistry, RCSI University of Medicine and Health Sciences, Dublin 2, Ireland; ‡ Dutch-Belgian Beamline (DUBBLE), ESRF - The European Synchrotron Radiation Facility, CS 40220, Grenoble 38043 Cedex 9, France; § Instituto de Química, Pontificia Universidad Católica de Valparaíso, Avda. Universidad 330, Curauma, Placilla 2950, Valparaíso, Chile; ∥ School of Pharmacy and Biomolecular Sciences and Tissue Engineering Research Group, RCSI University of Medicine and Health Sciences, Dublin 2, Ireland; ⊥ Science Foundation Ireland (SFI) Centre for Research in Medical Devices (CURAM), RCSI, Dublin 2, Ireland; # AMBER, The SFI Advanced Materials and Bioengineering Research Centre, RCSI, Dublin 2, Ireland

## Abstract

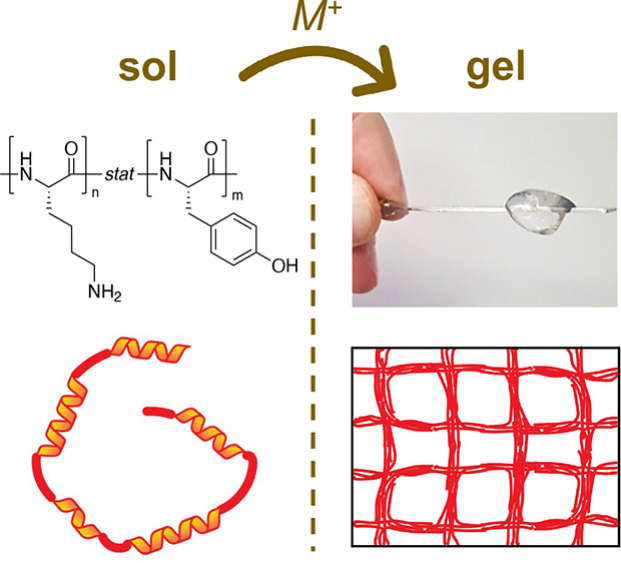

Statistical copolypeptides comprising lysine and tyrosine with unprecedented ion-induced gelation behavior are reported. Copolypeptides are obtained by one-step *N*-carboxyanhydride (NCA) ring-opening polymerization. The gelation mechanism is studied by in situ SAXS analyses, in addition to optical spectroscopy and transmission electron microscopy (TEM). It is found that the gelation of these statistically polymerized polypeptides is due to the formation of stable intermolecular β-sheet secondary structures induced by the presence of salt ions as well as the aggregation of an α-helix between the copolypeptides. This behavior is unique to the statistical lysine/tyrosine copolypeptides and was not observed in any other amino acid combination or arrangement. Furthermore, the diffusion and mechanical properties of these hydrogels can be tuned through tailoring the polypeptide chain length and ion strength.

Hydrogels comprise three-dimensional cross-linked networks, allowing them to hold significant amounts of water, which makes them attractive materials for biomedical applications such as drug delivery,^1^ wound dressing,^2^ antimicrobial coatings,^3^ and regenerative medicine.^4^ Natural hydrogels based on proteins (e.g., collagen, gelatin, and fibroin) are often the materials of choice due to their inherent biocompatibility. They form physical hydrogels through peptide-based biomacromolecule self-assembly.^5−8^ Although these natural polymers have drawn large attention from the biomaterials research community, the difficulty in their purification motivated researchers to explore synthetic peptide analogues. Two main synthetic approaches exist to design peptides capable of forming physical hydrogels.^9^ The first is based on the synthesis of peptides with precisely controlled amino acid sequences, of which several examples have been reported to form physical hydrogels either spontaneously or upon a trigger.^10−13^ While predominantly oligomeric, these peptides vary in length and share the common feature of forming self-assembled nanostructures through hydrophobic, ionic, hydrogen bonding, or secondary structure interaction.^14−16^ These typical higher-order motives include nanotubes, nanotapes, or nanospheres, which then aggregate to form hydrogel networks.

The second approach uses polymerization-derived polypeptides. The dominant design motive in these hydrogel-forming polypeptides is based on block structures.^17,18^ This includes hybrid block and graft copolymers containing a polypeptide and a synthetic block or pure polypeptide block copolymers. The latter are readily accessible by sequential amino acid *N*-carboxyanhydride (NCA) polymerization.^19,20^ Similar to oligopeptides, three-dimensional networks in aqueous media are formed through hydrophobic, ionic, as well as secondary structure interactions such as β-sheet- or α-helix-driven molecular arrangements.^21^ For example, Deming reported diblock copolypeptides incorporating oppositely charged ionic blocks that form β-sheet-structured hydrogel assemblies via polyion complexation.^22^ We and others have reported hydrogels through hydrophobic interaction from amphiphilic linear as well as branched block copolypeptides comprising lysine or glutamic acid in their hydrophilic blocks and phenylalanine, isoleucine, leucine, or alanine in their hydrophobic blocks.^23−25^

Statistical copolypeptides, which are copolymers obtained in a one-step binary copolymerization of NCAs, have not been proposed as hydrogelators to date, as it is indeed counterintuitive to assume their gelation due to the absence of defined blocks suitable for self-assembly. Here we disclose the first example of a statistical copolypeptide comprising lysine (Lys) and tyrosine (Tyr) that can form hydrogels upon addition of buffer salt solution. While for some natural proteins and sequence-defined oligopeptides salt-triggered gelation is known,^26,27^ salt usually compromises the stability of block copolypeptide hydrogels due to the destructive impact salts have on amphiphilic interaction.^28^ The statistical Lys/Tyr copolypeptides thus display properties otherwise only found in sequence-controlled oligopeptides, making them materials with an unprecedented structure/property profile. Through detailed characterization, we propose a unique mechanism explaining the salt-triggered transition from single copolypeptides to porous hydrogel networks. Moreover, preliminary physicochemical properties of the porous hydrogels (e.g., mechanical property and diffusion property) are discussed.

Two statistical copolypeptides, poly(l-lysine-*stat*-l-tyrosine), p(Lys_*x*_Tyr_*y*_), with total degrees of polymerization of 100 and 150 were synthesized from the protected monomers maintaining a fixed *N*-ε-carbobenzyloxy-l-lysine (l-Lys(Z)) to *O*-benzyl-l-tyrosine (l-Tyr(Bzl)) ratio of 4:1 (Scheme S1).^29^^1^H NMR analysis confirmed the copolypeptide composition (Figures S1 and S2), while size exclusion chromatography (SEC) showed monomodal traces with dispersities (*Đ*) around 1.2 and relative shifts in agreement with the targeted degrees of polymerization (Figure S5). After deprotection, no gelation was observed in DI water for any of these two copolypeptides. After mixing with phosphate-buffered saline (PBS) solutions of different concentrations, both copolypeptides were found to form self-supporting hydrogels over time at a minimum copolypeptide concentration of 1.9 mM (Table 1, Figure 1). Furthermore, mixing of the copolypeptide DI water solution with CaCl_2_ or MgCl_2_ induced rapid yet heterogeneous gelation, suggesting that the gelation mechanism of these copolypeptides is induced by the presence of charged ions (e.g., Na^+^ in PBS buffer). For the following studies, PBS solution, due to its weak ion strength, was chosen for initiating the gelation to avoid the morphological inhomogeneity caused by rapid gelation (concentration: 2.5 and 3.8 mM for p(Lys_120_Tyr_30_) and p(Lys_80_Tyr_20_)).

**Figure 1:**
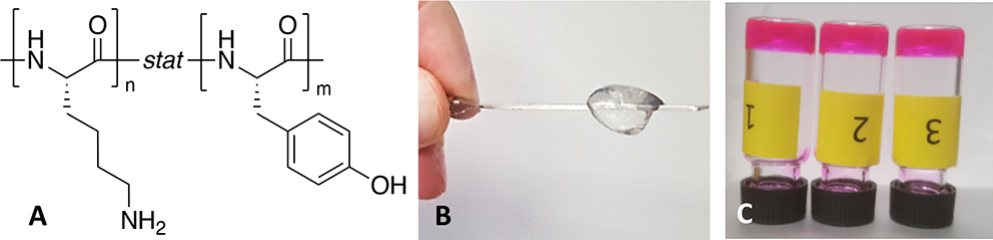
Structure of statistical copolypeptide poly(l-lysine-*stat*-l-tyrosine) (A), image of hydrogel in PBS buffer on a spatula (B), and examples of hydrogels dyed with Rhodamine B.

**Table 1:** Dependence of p(Lys_80_Tyr_20_) Gelation (Vial Inversion) on Copolypeptide and PBS Buffer Concentration

	PBS concentration			
*M* (copolypeptides)	150 mM	100 mM	50 mM	25 mM
1 mM	no gel	no gel	no gel	no gel
1.9 mM	gel	gel	gel	no gel
3.8 mM	gel	gel	gel	no gel
7.6 mM	gel	gel	gel	no gel

To elucidate the mechanism of this unusual gelation, the hydrogel sample composed of p(Lys_80_Tyr_20_) formed in PBS D_2_O was first analyzed by FTIR spectroscopy. As shown in Figure 2a, a predominant peak can be found at ca. 1624 cm^–1^ in the polypeptide hydrogel’s amide I band, indicating the formation of antiparallel β-sheet secondary structures in the copolypeptide hydrogel sample.^30−32^ An additional weak band near 1680 cm^–1^ originating from this antiparallel β-sheet structure can also be observed.^33^ On the other hand, a strong band near 1646 cm^–1^ suggests the presence of α-helical secondary structures, which is quite common for polylysine in basic solution.^34^ Notably, it has been reported that Tyr-Lys pairs can stabilize the helical structure.^35^ Interestingly, no observable β-sheet secondary structure can be found in this copolypeptide’s nongelling D_2_O solution in the absence of salt ions. Instead, in addition to the absorption belonging to the α-helix structure, a strong IR band appeared near 1671 cm^–1^, indicating a dominating secondary “β-turn” structure.^36^ Circular dichroism analyses further supported the FTIR findings (Figure 2b). The minimum at 217 nm for the polypeptide gel formed 2 h after mixing with PBS suggests predominant antiparallel β-sheet structures,^37^ while the two minima at 207 and 220 nm observed in the CD spectrum of the H_2_O solution alone are indicative fingerprint minima of the α-helix secondary structure in polypeptides.^38^

**Figure 2:**
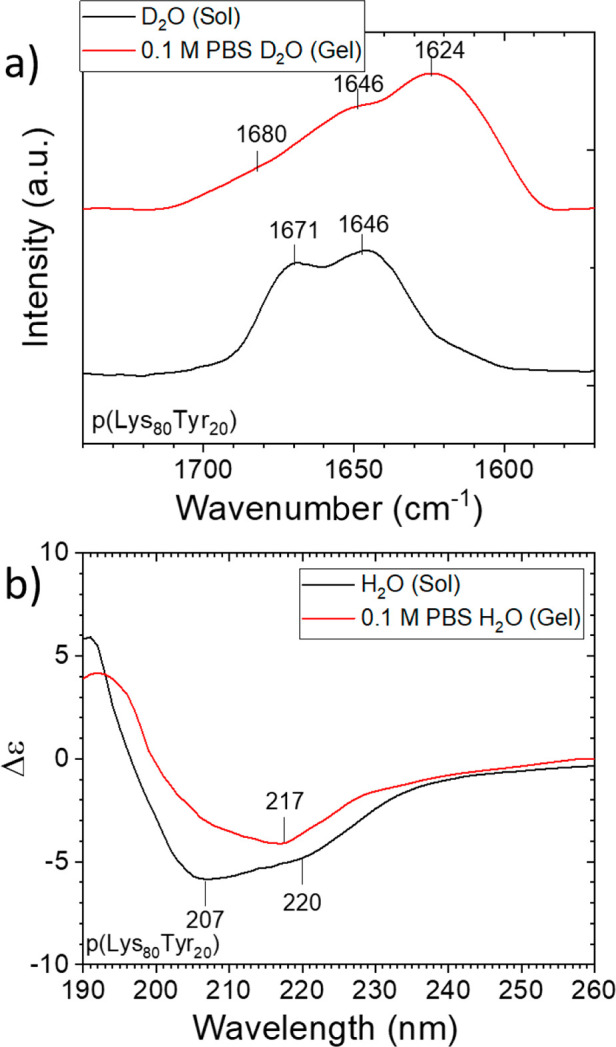
(a) FTIR analyses of poly(lys_80_-tyr_20_) recorded in D_2_O and 2 h after mixing with 0.1 M PBS buffer D_2_O solution (for deconvolution, see Figure S9). (b) CD analyses of poly(lys_80_-tyr_20_) recorded in H_2_O and 2 h after mixing with 0.1 M PBS buffer H_2_O solution.

Next, the p(Lys_80_Tyr_20_) polypeptide gelation process was investigated by fluorescence spectroscopy. The spectrum of the polypeptide in 0.1 M PBS buffer solution taken after 2 h of mixing showed a significant increase in the intensity of the peak around 305 nm compared to the same copolypeptide in water, while a broad peak around 400 nm remained constant after the mixing (Figure S6). The former fluorescence emission is characteristic of the tyrosine units of the polypeptides, while the latter belongs to the aggregated lysine residues.^39^ Similar observations were made for both samples. Since the relative monomer composition of the two copolypeptides was kept constant during the measurement, this strengthening in intensity can only be caused by a change in the local environment of the lysine residues.

As suggested by previous studies,^40^ when a stable localized intramolecular H bonding can be formed between tyrosine units (e.g., α-helix structure), the local environment for the tyrosine unit becomes more hydrophobic. This hydrophobicity may induce the formation of specific tertiary structures that result in colocalization of the tyrosine residues and prevent the observation of fluorescence emission of these tyrosine units.^41^ On the other hand, it has been reported that metal ions can effectively unfold a natural peptide molecule and promote the formation of tyrosine-based intermolecular β-sheet structure.^42,43^ Hence, one can speculate that in the presence of PBS buffer (namely, Na^+^ and K^+^) the tertiary structures of the copolypeptides are disturbed and subsequently unfold (reduction of the internal α-helix structure). This unfolding does result in the exposure of tyrosine units possible, while the subsequent formation of the intermolecular β-sheet induces the gelation of these polypeptides.

To obtain more insight into the morphological changes during the gelation process, a series of in situ SAXS analyses were carried out on the hydrogels. Here we used a flexible worm-like polymer chain model to fit our system as suggested by previous studies on peptide-based self-assembly systems.^44−47^ As shown in Figure 3a, for samples containing p(Lys_80_-Tyr_20_) mixed with 0.1 M PBS buffer, all scattering profiles can be fitted with a combination of two worm-like polymer chain form factor models with a structure factor of a mass fractal object (for a detailed fitting procedure description, see the SI). This type of form factor model has been frequently applied to fibrous hydrogel networks,^47,48^ while the fractal structure factor has also been used for branched fibrous hydrogel structures self-assembled from short polypeptides.^45^ This model is supported by the observation of branched fibrous structures in the TEM analyses of polypeptide samples mixed with PBS buffer (Figure S7). Figure 3b shows the evolution of the fitted fractal dimension (*D*_f_) over the gelation period. The whole gelation can be described as a process of a gradual branching of the network structure. Furthermore, it can be found that higher ion concentration does increase the branching rate and overall fractal dimension of the material. A similar effect of ion concentration on the nanofiber has also been observed in several short oligopeptide nanoassembly systems.^44^ Additionally, the elevated PBS concentration can also increase the gelation speed.

**Figure 3:**
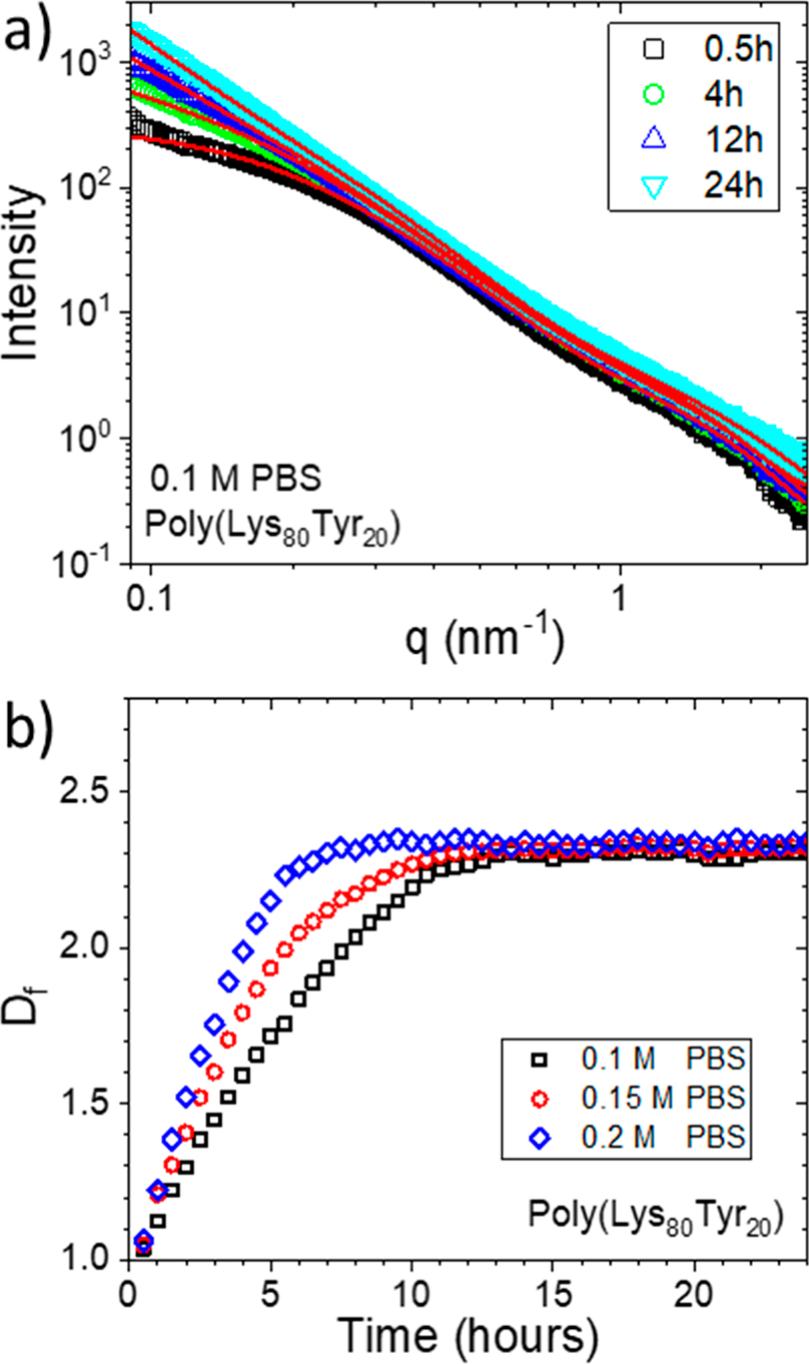
(a) Scattering profiles of the solution composed by 3.8 mM poly(Lys_80_Tyr_20_) and 0.1 M PBS buffer over a 24 h period. The model fitting is represented by the red line. (b) The fitted fractal dimension (*D*_f_) of a 3.8 mM poly(Lys_80_Tyr_20_) sample mixed with different concentrations of PBS solution over a period of 24 h.

Based on these results, we propose the following gelation model for this type of random copolypeptide. As shown in Figure 4a, due to the presence of charged ions, the α-helix structural domains of the copolypeptide formed by Lys and Tyr units is disrupted. Furthermore, as previously only observed in the micellar assemblies formed by those sequence-defined oligopeptides,^27^ these charged salts can induce a conformational transition from the α-helix to intramolecular β-sheet. These β-sheet units can subsequently interact with β-sheet domains in adjacent copolypeptides to form intermolecular β-sheets. As a result of the gradual increase in the number of the intermolecular connection between individual polypeptides, a worm-like bundle structure is produced over time (Figure 4b). Finally, when a critical concentration of copolypeptides and ions was used, a hyper-branched network was formed by the end of the gelation (Figure 4c). To the best of our knowledge, a gelation process through salt-triggered secondary structure transition has never been described for any copolypeptide. It appears to be a unique characteristic of the Lys/Tyr statistical copolypeptides, as neither the Lys/Tyr block copolypeptide nor statistical copolypetides in which Lys was replaced by glutamic acid (Glu) or Tyr by phenylalanine (Phe) result in any gelation. Additional preliminary screening of different copolypeptide compositions revealed that a minimum of 9% Tyr (mol/mol) in the copolymer is needed to obtain hydrogels in PBS buffer (Table S1). All hydrogels were found to be stable against dilution with DI water as well as over two months at 37 °C. Addition of trifluoroacetic acid (TFA) causes the hydrogels to dissolve. Further systematic experiments will be necessary to fully understand the influence of the monomer arrangement in the copolypeptides. This includes the elucidation of preferential monomer addition, potentially resulting in segments rich in one monomer.

**Figure 4:**
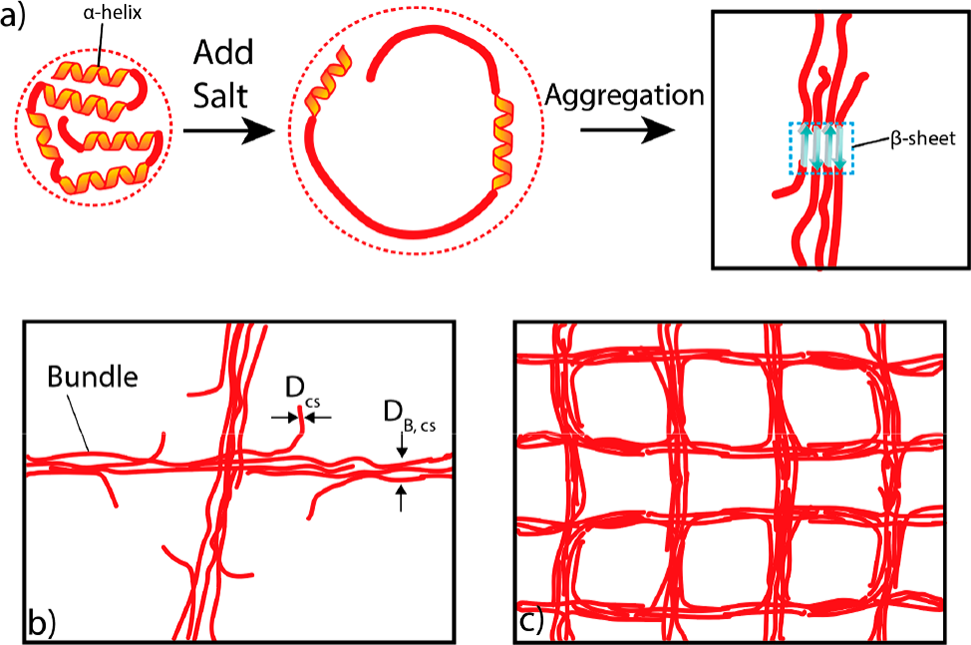
Illustration for the gelation process. (a) The introduction of salts disrupts the quaternary structure of the polymer and increases the hydrodynamic size of the polymer in the solution. When the concentration of the polymer is over a certain threshold, the intermolecular β-sheet starts forming. (b) A worm-like “bundle” structure was formed over time; *D*_cs_ is the diameter of the free polymer chain end; and *D*_cs,B_ is the diameter of the bundle structure. (c) When a proper polypeptide and ion concentration are used, a superporous network structure can be finally produced.

The fully hydrogelated samples were further investigated in a series of scattering, mechanical, and diffusion tests to understand their macroscopic physical properties in relation to their microscopic structure. Six polypeptide networks were produced from the two polypeptides p(Lys_120_Tyr_30_) and p(Lys_80_Tyr_20_) in combination with three different PBS concentrations. As shown in Figure 5a, the final gel state of these networks can all be well fitted with a worm-like polymer bundle model. It also can be found that the hydrogels made from the higher molecular weight copolypeptides have generally smaller cross-section diameters (*D*_cs,B_) for their bundle structure while obtaining a larger *D*_f_ (Figure 5b). Since these hydrogel network structures are induced by the intermolecular β-sheet formation (and aggregation of the α-helix to smaller extent), this correlation suggests that fewer copolypeptides are participating in the formation of the individual bundle structure for polypeptides with higher molecular weight. Furthermore, the ion concentration seems to have a negative impact on the cross-section’s diameter of these network structures, as a smaller cross section was observed in copolypeptide networks formed at higher ion concentration. This may arise from the drop in the Debye screening length (*L*_κ_) at higher ionic strength, which can result in a better bundle compactness. Similar observations were reported for short oligopeptide nanoassemblies.^44^ Additional NMR diffusometry and mechanical tests also show that the physical properties of these hydrogels can be tuned through varying the salt concentration as well as the copolypeptide chain length. Pulsed field gradient NMR^49,50^ was used to analyze the self-diffusion coefficients of H_2_O, , in these hydrogels. As shown in Figure 5c, due to the high water content and porous nature of these hydrogels, all the diffusion curves can be fitted with a single-component exponential decay function, and the extracted  values for all the hydrogels can be found with the same magnitude as the value of the free diffusing water in PBS buffer solution (). Further analyses on these  values across all the samples revealed that the ionic strength of the solution does not play a significant role in the diffusion of small solutes in these hydrogels since similar slopes can be found for the linear fittings of these values upon increasing the PBS concentration across the copolypeptide hydrogels with different chain length (Figure 5d). However, an observable change can be found for hydrogels made from copolypeptides with different chain length with slightly slower water diffusion in the hydrogel from a higher molecular weight, which may arise from the decrease in the mesh size of the networks as indicated from a larger *D*_f_ value for samples containing poly(Lys_120_Tyr_30_). While the materials are considered soft gels from the rheological analysis (Figure 5e), smaller mesh sizes also increase their mechanical properties as shown in their storage modulus analyses (Figure 5f).

**Figure 5:**
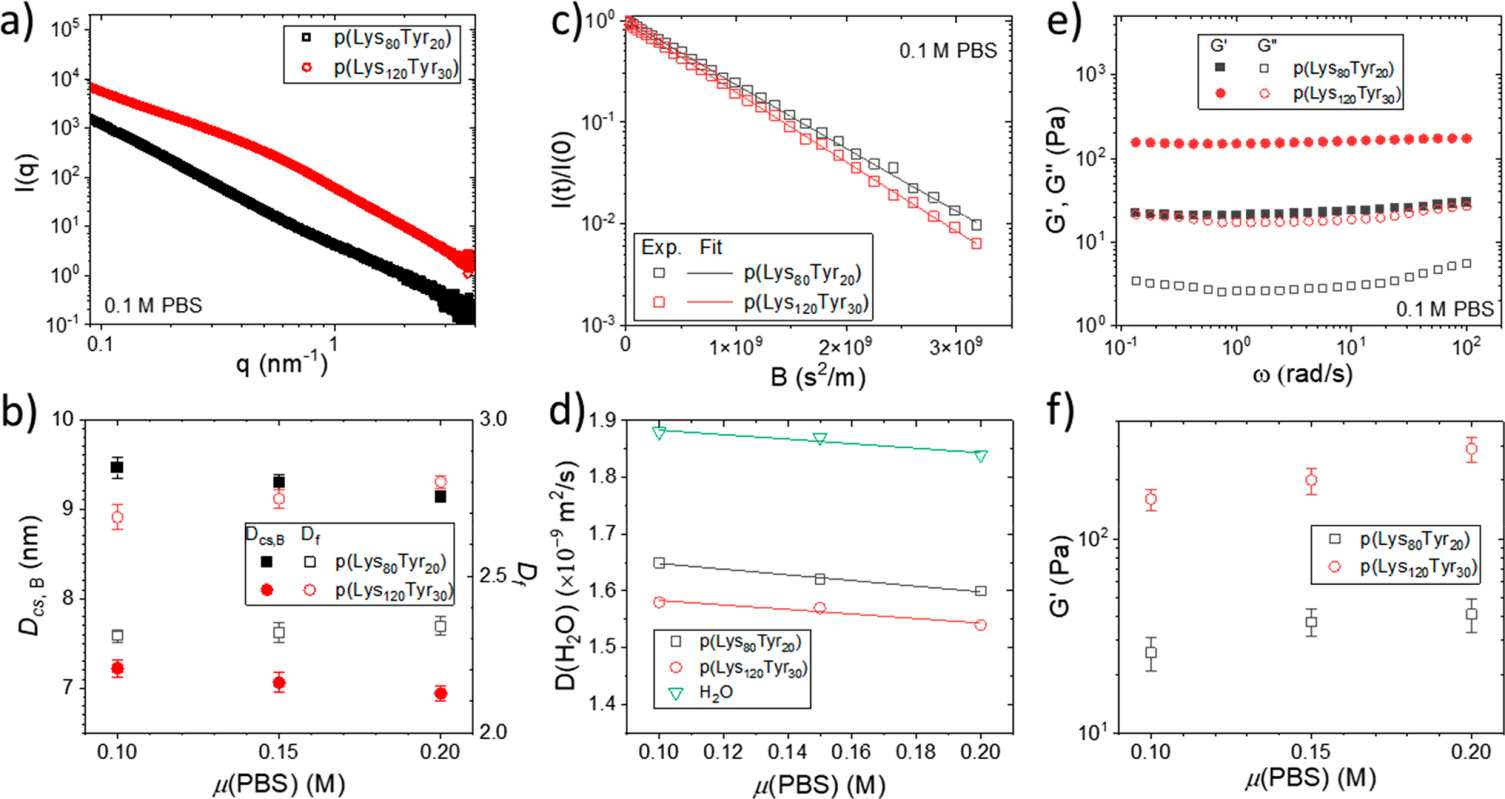
(a) Scattering profiles of the final gel composed by 0.1 M PBS buffer solution and 2.5 mM poly(Lys_120_Tyr_30_) and 3.8 mM poly(Lys_80_Tyr_20_). These scattering profiles are elevated proportionally for better display and comparison. (b) The extracted cross-section diameters (*D*_cs_) and the fracture dimension (*D*_f_) of the network structure in the final hydrogels with different compositions. All these profiles are recorded after 7 days of incubation to ensure the completion of the gelation. (c) ^1^H NMR diffusometry analyses of H_2_O diffusion in hydrogels made from different polypeptides mixed with 0.1 M PBS. (d) Diffusion coefficients of H_2_O, , in solutions and different hydrogels. (e) Frequency sweep of hydrogels made from polypeptides mixed with 0.1 M PBS. (f) Plot of storage moduli (*G*′) of the hydrogel samples versus their PBS concentrations.

In summary, we have demonstrated the first example of an ion-responsive hydrogellating statistical copolypeptide. A simple but unique gelation mechanism is proposed that relies on the ion-triggered structural rearrangement of the polypeptides to transition from intramolecular to intermolecular secondary structures. The fact that these porous hydrogels form in physiological buffer solution offers opportunities in biological application, for example, through incorporation of cells. Future work will focus on testing more variations of these statistical copolypeptides to better understand various factors’ contribution to their gelation mechanism as well as their investigation as biofunctional materials.

## Supporting Information Available

Experimental procedures and additional data (PDF)
