# Supplementary material for: Ion-Triggered Hydrogels Self-Assembled from Statistical Copolypeptides
Source: ACS Macro Lett. 2022 Feb 16;11(3):323–8. doi: 10.1021/acsmacrolett.1c00774 (PMC8928472; doi:10.1021/acsmacrolett.1c00774)
Supplement: Supplementary file 1 — mz1c00774_si_001.pdf [file mz1c00774_si_001.pdf]

Ion-triggered nanoporous hydrogel self-assembled from randomly copolymerized polypeptides

Bing Wu,[a,b]* Saltuk B. Hanay,[a] Scott D. Kimmins,[c] Sally-Ann Cryan,[d,e,f] Daniel Hermida Merino,[b] Andreas Heise.[a,e,f]*

a. Department of Chemistry, RCSI University of Medicine and Health Sciences, Dublin 2, Ireland.
b. Dutch-Belgian Beamline (DUBBLE), ESRF- The European Synchrotron Radiation Facility, CS 40220, 38043 Grenoble Cedex 9, France
c. Instituto de Química, Pontificia Universidad Católica de Valparaíso, Avda. Universidad 330, Curauma, Placilla, Valparaíso, Chile
d. School of Pharmacy and Biomolecular Sciences and Tissue Engineering Research Group, RCSI University of Medicine and Health Sciences, Dublin 2, Ireland
e. AMBER, The SFI Advanced Materials and Bioengineering Research Centre, RCSI, Dublin D02, Ireland
f. The SFI Centre for Medical Devices (CURAM) RCSI University of Medicine and Health Sciences 123 St Stephen's Green, Dublin 2, Dublin (Ireland)

Corresponding authors: friedrichbing.wu@utoronto.ca ; andreasheise@rcsi.ie

1 Experimental Procedures

1.1 Materials

All reagents were purchased from Sigma-Aldrich unless otherwise noted. Phosphate-buffered saline (PBS) tablets was used to prepare the PBS solution. Protected amino acids were purchased from BACHEM. Triphosgene was purchased from Fluorochem. *N*-ε-Carbobenzyloxy-L-lysine (L-Lys(Z)) and *O*-Benzyl-L-tyrosine (L-Tyr(Bzl)) were converted to the corresponding NCAs using triphosgene as described in literature.[1] *N*,*N*-Dimethylformamide, 99.8%, extra dry over Molecular Sieve (AcroSeal™ was purchased from ACROS Organics).

Synthesis of copolypeptides; example of poly(LLys$_{80}$-*stat*-LTyr$_{20}$): Poly(LLys(Z)$_{80}$-*st*-LTyr(Bz)$_{20}$) was synthesized as previously reported.[2] 1.09 g (3.56 mmol) LLys(Z) NCA and 267 mg (0.89 mmol) of

LTyr(Bz) NCA were dissolved in 10 mL anhydrous DMF and the solution was purged with nitrogen for 10 min. 9 mg hexylamine (1.8 mL of 50 mg mL^{-1} stock solution in DMF ~ 0.089 mmol) was added fast to the solution and stirred under high vacuum at room temperature. The reaction was monitored by FTIR spectroscopy until all monomer was consumed (disappearance of NCA bands at 1780 and 1850 cm^{-1}) (6–8 h). Then, the copolypeptide solution was precipitated dropwise into 200 mL diethyl ether. After decanting the solvent, fresh diethyl ether was added and stirred for 20 min (2 times). The product was then collected and dried under vacuum. Yield: 1.08 g, 93%. ^{1}H-NMR analysis was in agreement with previous results.[2]

Copolypetide deprotection: 600 mg of poly(LLys$(Z)_{80}$ -*st*-LTyr$(Bz)_{20}$) was dissolved in 5-6 mL trifluoroacetic acid. To this solution, 0.5 mL of HBr (33% in acetic acid) was added dropwise and stirred overnight. Then, the solution was precipitated in 100 mL diethyl ether. After the product was collected, it was washed with an excess diethyl ether and dried under vacuum. The dried polymer was dissolved in water and placed into a 3.5 MWCO snake skin dialysis tubing and dialyzed against water for 3 d (refreshing water 4–5 times a day). Finally, the solution was freeze dried and white fluffy product collected. Yield: 410 mg, 89% (see Figure S1 for ^{1}H-NMR spectrum, S2 for ^{13}C NMR spectrum and S5 for SEC results).

Scheme S1. Synthesis of statistical copolypeptides poly(L-lysine-*st*-L-tyrosine).

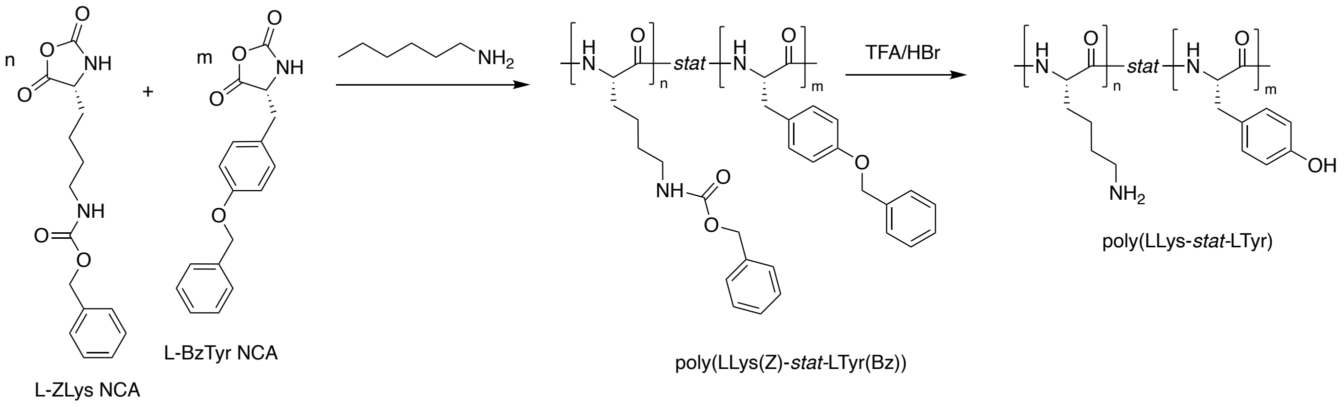

Table S1: Additional statistical copolypeptides and their gelation behaviour.

	PBS concentration			
Polypeptide composition and concentration (w/w)	150 mM	100 mM	50 mM	25mM
Lys_{91}-*stat*-Tyr_{9} (5 %)	no gel	no gel	no gel	no gel
Lys_{91}-*stat*-Tyr_{9} (10 %)	gel	no gel	no gel	no gel
Lys_{85}-*stat*-Tyr_{15} (5 %)	gel	gel	-	-
Lys_{85}-*stat*-Tyr_{15} (10 %)	gel	gel	-	-

Lys_{40}-*stat*-Tyr_{10} (5 %)	gel	no gel	-	-
Lys_{40}-*stat*-Tyr_{10} (10 %)	gel	no gel	-	-
Lys_{100} (10%)	no gel	no gel	no gel	no gel
Tyr_{100} (10%)	no gel	no gel	no gel	no gel
Glu_{80}-*stat*-Tyr_{20} (10%)	no gel	no gel	-	-
Lys_{70}-*b*-Tyr_{30} (10%)	no gel	no gel	no gel	no gel
Lys_{70}-*stat*-Tyr_{30} (10%)	gel	gel	gel	gel

1.2 Methods

Attenuated total reflection (ATR) FT-IR analyses: Attenuated total reflection (ATR) FT-IR spectra were recorded using a Perkin-Elmer Spectrum 100 in the region of 4000-650 cm^{-1}. 8 scans were completed with a resolution of 2 cm^{-1}. A background measurement was performed prior to loading the sample onto the ATR for measurement. For gelation analyses, a proper solvent subtraction is performed via using D_2O as substitute to reduce the overlapping between H_2O's O-H stretching band and amide's N-H stretching band.

Size Exclusion Chromatography (SEC): SEC was carried out in 1,1,1,3,3,3-Hexafluoro-2-propanol (HFIP) using a PSS SECurity GPC system equipped with a PFG 7 µm 8 × 50 mm pre-column, a PSS 100 Å, 7µm 8 × 300 mm and a PSS 1000 Å, 7µm 8 × 300 mm column in series and a differential refractive index (RI) detector at a flow rate of 1.0 mL min^{-1}. The SEC system was calibrated against Agilent Easi-Vial linear poly(methyl methacrylate) (PMMA) standards and analysed by PSS winGPC UniChrom software package.

Rheology Analysis: Rheological measurements were conducted on an MCR 301 digital rheometer (Anton Paar, Physica, Australia). Frequency sweeps (1–100 rad/s and $\gamma = 0.1\%$), strain sweeps ($\gamma = 0.1–100\%$ and 1 rad/s), and time sweeps with oscillation in strain (stepping between 0.1 and 40%, then 0.1 and 100%, and returning to 0.1% strain at 1 rad/s) were conducted at room temperature (21 °C) using a conical plate (CP50-1, Anton Paar, Australia) consisting of a 50 mm diameter geometry and a 1° cone angle with a gap length of 0.097 mm. The use of a protective hood was employed to prevent evaporation.

Circular Dichroism Spectroscopy: CD data were collected on a Jasco J-810 CD spectrometer (Japan Spectroscopic Corporation) with a path length of 0.1 cm and a bandwidth of 1 nm. Three scans were conducted and averaged between 185 and 350 nm at a scanning rate of 20 nm min^{-1} with a resolution of 0.2 nm. The data were processed by subtracting solvent as background.

Electron Microscopy: TEM image were recorded on a Hitachi H-7650 instrument. The sample (5 μL) were dropped on a Cu grid coated with Formvar and were wiped off after 30 s. Scanning electron microscopy (SEM) images were recorded in a Shimadzu SSX-550 instrument. The samples were coated with gold using a vacuum sputter coating system to improve their electrical conductivity and thus the quality of the images, which were recorded using secondary electrons.

Synchrotron-based SAXS analyses: Synchrotron-based small-angle X-ray scattering (SAXS) measurements were performed on BM26B (DUBBLE) at the European Synchrotron Radiation Source, Grenoble, France. The sample to SAXS detector distance was about 3 m using a wavelength λ = 0.9994 Å. A Dectris-Pilatus 1 M detector with a resolution of 981 × 1043 pixels and a pixel size of 172 × 172 μm was employed to record the 2D SAXS scattering patterns. Standard corrections for sample absorption and background subtraction have been performed prior to the data reduction. The scattering data were further normalized to the intensity of the incident beam (to account for primary beam intensity fluctuations). The scattering pattern from AgBe was used for the calibration of the wavenumber ($q = 4\pi sin\theta/\lambda$) scale of the scattering curve. The sample was placed in a quartz capillary (d = 2 mm) and kept at 20 °C using a Linkam stage. SAXS data frames were acquired each 30 min during the gelation process. The SAS instrument has a pinhole geometry and the high flux of the X-ray beam from the synchrotron reduced the data collection time down to 2 s, thus allowing the use of SAXS to monitor the gelation process in real time. Absolute intensities were obtained using the secondary standard method with pure water.[3]

SAXS Model fitting was performed by the SASFit software.[4] The scattering of polymers in solution could be described using a wormlike chain model proposed by Kholodenko.[5] The scattering intensity in this model can be described as the following equation:

$$I(q) = (\Delta\rho)^2 \phi P_0(q, L, 2l_p) P_{cs}(q, D_{cs}) \qquad (S1)$$

Where $\Delta\rho = \rho_{polymer} - \rho_{solvent}$ is the electron density difference between polymer chain and the solution, ϕ is the polymer volume fraction, L is the chain contour length, l_p is the polymer chain persistence length (half of the Kuhn length), and the D_{cs} is cross-sectional diameter of the polymer chain.

The scattering from the network architecture was described using a combination of two wormlike models, one represents the polymer bundle structure, while the other was added to account for the re-dissolved polymer ends and small polymers:

$$\begin{aligned} I(q) &= I_{bundles}(q) + I_{polymers}(q) \\ &= (\Delta\rho)^2 \phi P_0(q, L_B, 2l_{p,B}) P_{cs}(q, D_{cs,B}) + (\Delta\rho)^2 \phi P_0(q, L, 2l_p) P_{cs}(q, D_{cs}) \qquad (S2) \end{aligned}$$

where L_B is the polymer contour length, $l_{p,B}$ is the persistence length of the polymer bundle, $D_{CS,B}$ is the cross-sectional diameter of the bundles, L, l_p and D_{CS} are the parameter set for small polymers or dangling polymer ends.

In this study, due to the branched nature of the hydrogel network, we also adopted a fractal object based structure factor for the scattering from bundles-like structure[1],

$$S(q) = \frac{\sin[(D_f - 1)tan^{-1}(q\xi)}{(D_f - 1)q\xi(1 + q^2\xi^2)^{(D_f-1)/2}} \qquad (S3)$$

Where, D_f is the fractal dimension, $\xi^2 = 2R_g^2/(D_f(D_f + 1))$, and R_g is the radius of gyration of the fractal aggregate. Hence the total scattering intensity can be fitted with

$$I(q) = I_{bundles}(q)S(q) + I_{polymers}(q)$$
$$= (\Delta\rho)^2\phi P_0(q, L_B, 2l_{p,B})P_{cs}(q, D_{cs,B})\frac{\sin[(D_f - 1)tan^{-1}(q\xi)}{(D_f - 1)q\xi(1 + q^2\xi^2)^{\frac{D_f-1}{2}}} + (\Delta\rho)^2\phi P_0(q, L, 2l_p)P_{cs}(q, D_{cs})$$
$$(S4)$$

Because L_B of bundles is larger than the SAXS resolution, we kept L_B fixed at 970 nm. Additionally, to reduce the amount of fitting parameters and considering that the persistence length of the bundles is relatively large and does not have a major effect on the model in the fitted q range, we fixed $l_{p,B}$ to 270 nm for all the gels. We also assume R_g, L, l_p and D_{CS} does not change throughout the gelation process. The individual contributions of the models, D_f and $D_{CS,B}$ were fitted. The error of the bundle size $D_{CS,B}$ and fractal dimension D_f was determined after fitting the scattering curves, by changing the value until the fit did not converge anymore. Fig. S8 shows an example of the fitting with individual component displayed separately.

Nuclear Magnetic Resonance (NMR) Analyses: All NMR experiments were performed on 11.7T Bruker Avance III NMR spectrometer equipped with a four channel (^1H, ^{13}C, ^{15}N, ^2H) 4 mm CMP MAS probe, fitted with actively shielded gradient. All samples were swollen in D_2O, and kept locked by deuterium throughout the whole experiment. The spinning rate of the rotor was kept at 6 kHz. Inverse-gated decoupling pulse sequences were used in this study to record ^{13}C NMR spectrum. The 90° pulse length for ^{13}C channel was calibrated to 6.5 μs. Other acquisition parameters are following: 1) 16k acquisition points; 2) 4s recycle delay; 3) 4096 scans; 4) 50 kHz spectral width. The ^1H DOSY (Diffusion Ordered Spectroscopy) experiment was performed using the pulsed-field gradient (PFG) spin-echo (SE) sequence with phase cycling of radio frequency pulses and a spoil gradient to suppress artifacts. A total of 32 increments were collected for the DOSY dimension with 32 transients collected for each increment. The

gradient was ramped in a linear fashion from 2 to 98% of full the gradient strength 30 G/cm/A. Two sine shaped gradient pulses of 1.1 ms duration was used for both coding and decoding. Typical acquisition parameters are as following: 1) 16k acquisition points 2) 2.0s recycle delay and 200ms diffusion time 3) 10 kHz spectral width. All the NMR measurements were carried out at 20 °C.

2. Additional data

NMR analyses of copolypeptide structure

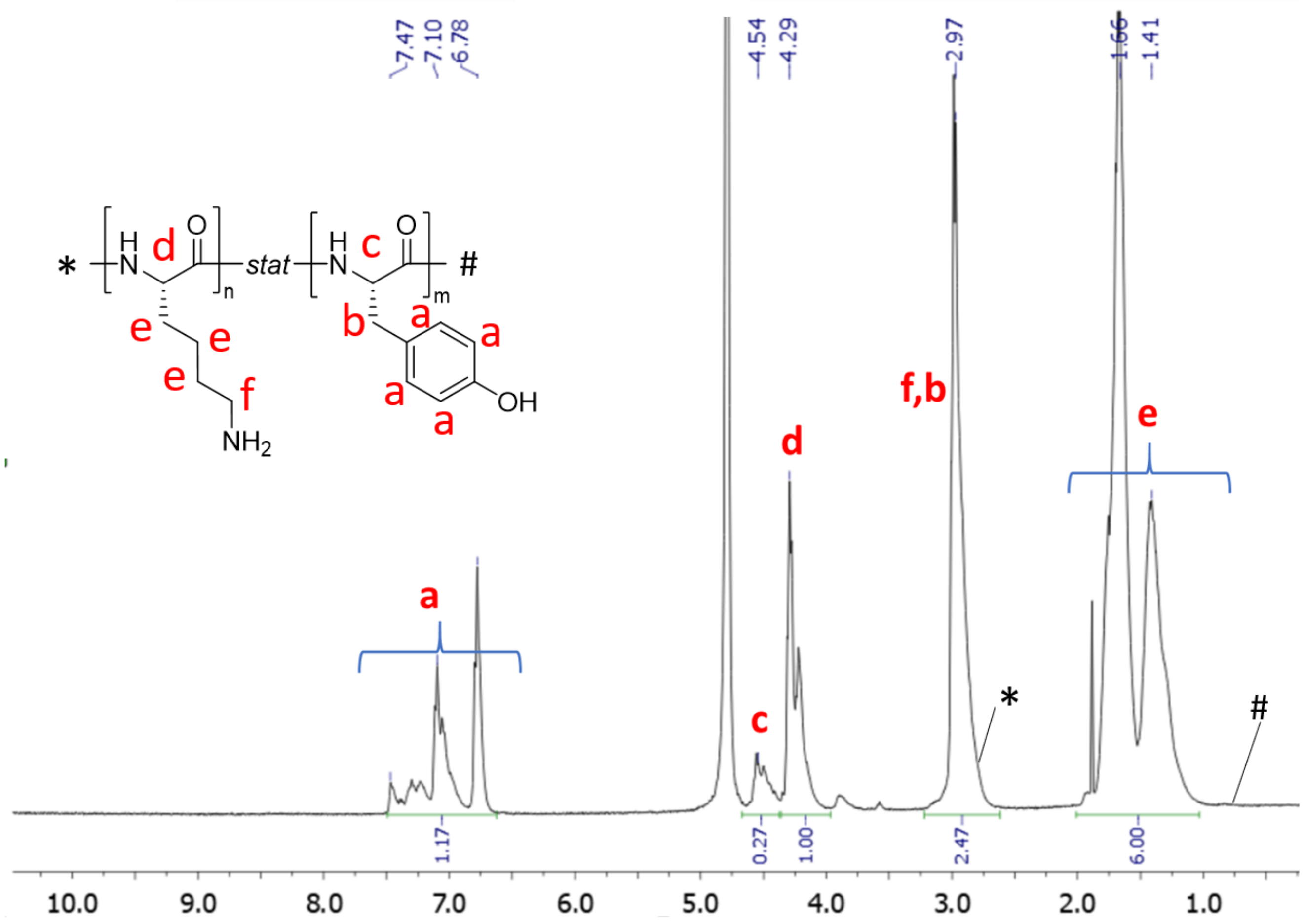

Figure S1. ^{1}H NMR spectrum of $p(Lys_{80}Tyr_{20})$ sample in D_2O with assignment.

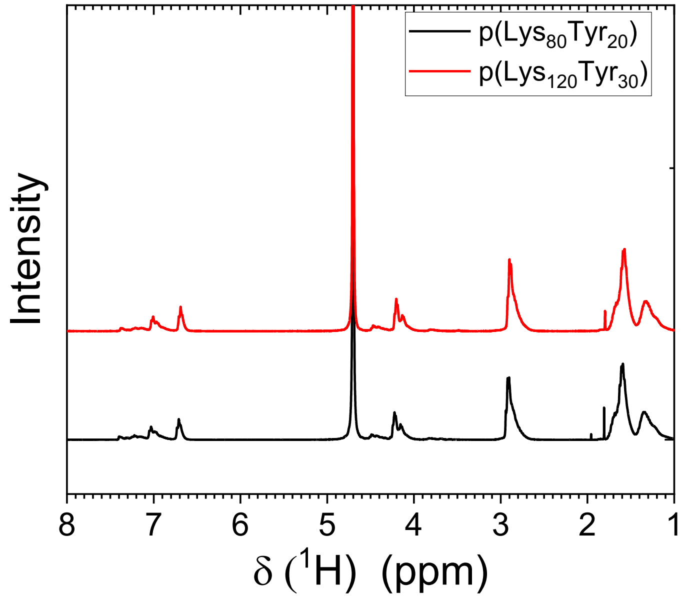

Figure S2. ^{1}H NMR spectra of $p(Lys_{80}Tyr_{20})$ and $p(Lys_{120}Tyr_{30})$ in D_2O.

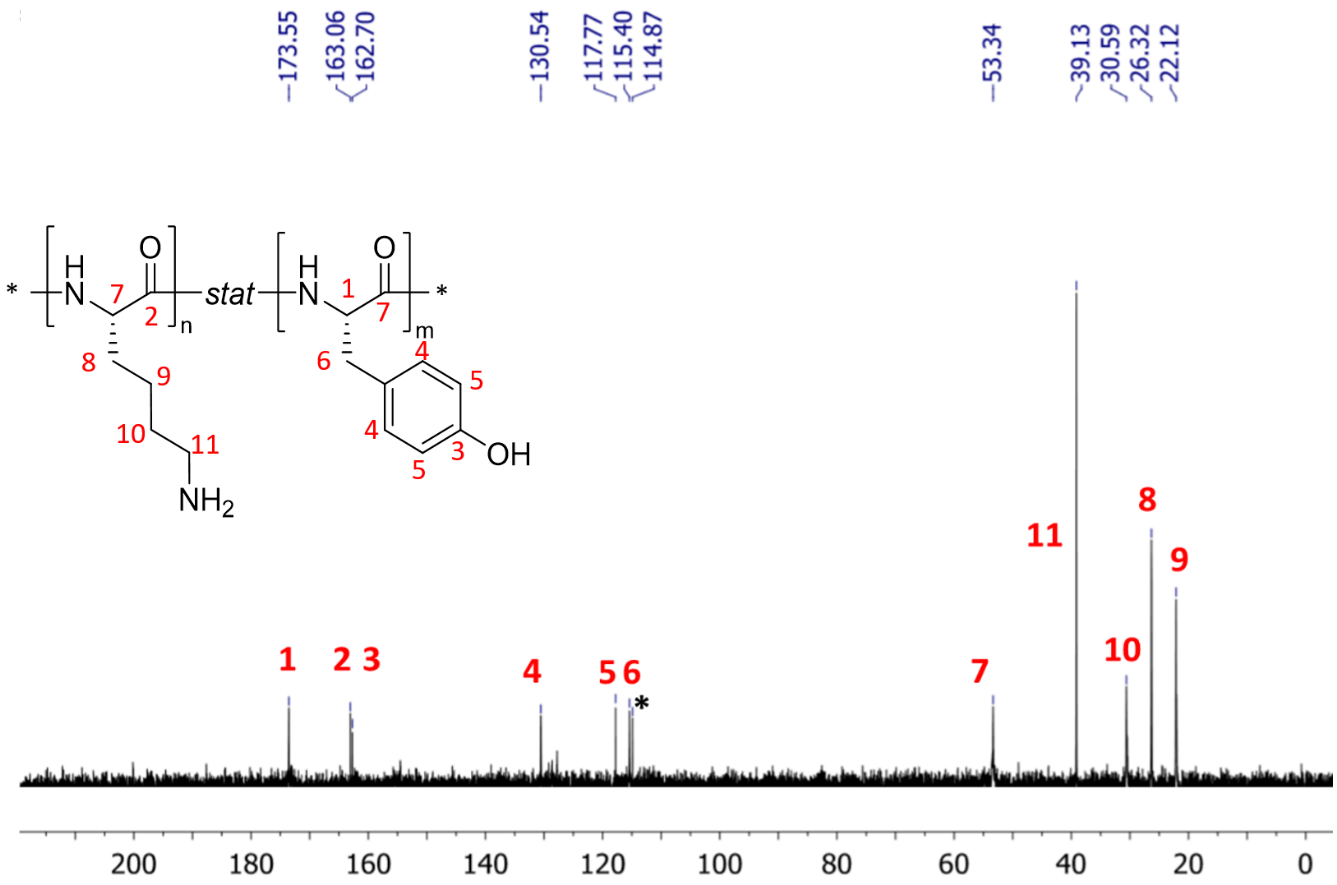

Figure S3. ^{13}C-NMR spectrum of p($Lys_{80}Tyr_{20}$) sample in D_2O.

FTIR analyses of dry polymer.

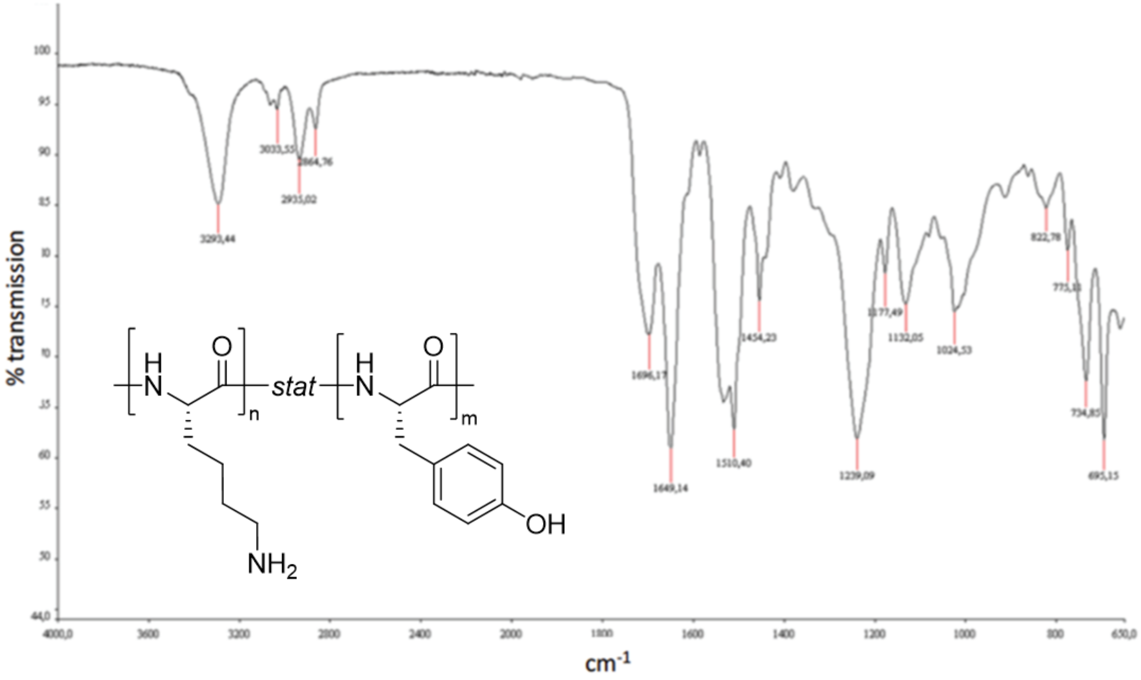

Figure S4. ATR FTIR spectrum of dry p($Lys_{80}Tyr_{20}$) sample.

Gel Permeable Chromatography Analyses

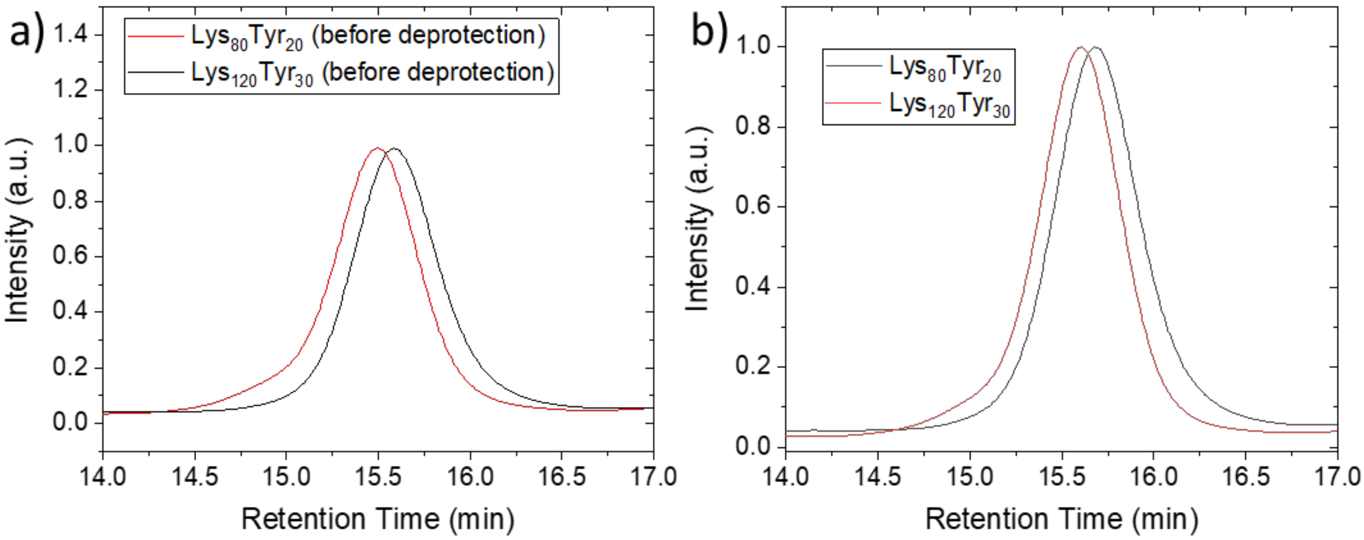

Figure S5. Statistical copolymer before and after deprotection. a) SEC analyses of poly(Lys(Z)$_{80}$-*stat*-LTyr(Bz)$_{20}$) and poly(LLys(Z)$_{120}$-*st*-LTyr(Bz)$_{30}$). b) SEC analyses of poly(LLys$_{80}$-*stat*-LTyr$_{20}$) (M_n 20,200 g/mol, $Đ$ 1.2) and poly(LLys$_{120}$-*st*-LTyr$_{30}$) (M_n 13,800 g/mol, $Đ$ 1.2).

Fluorescence Spectroscopy Analyses

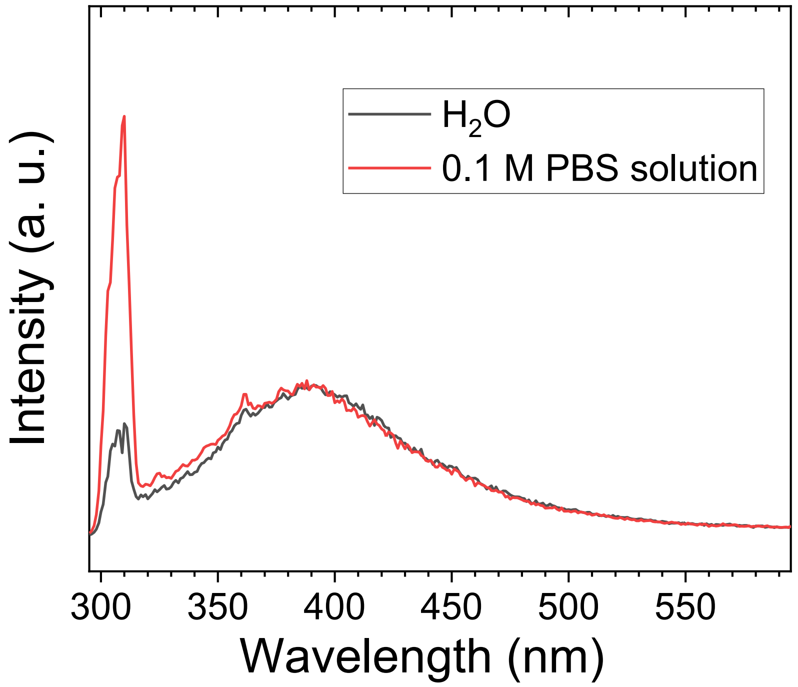

Figure S6. Fluorescence spectroscopy of $p(Lys_{80}Tyr_{20})$ H_2O and 0.1 M PBS solution.

Electron Microscopy:

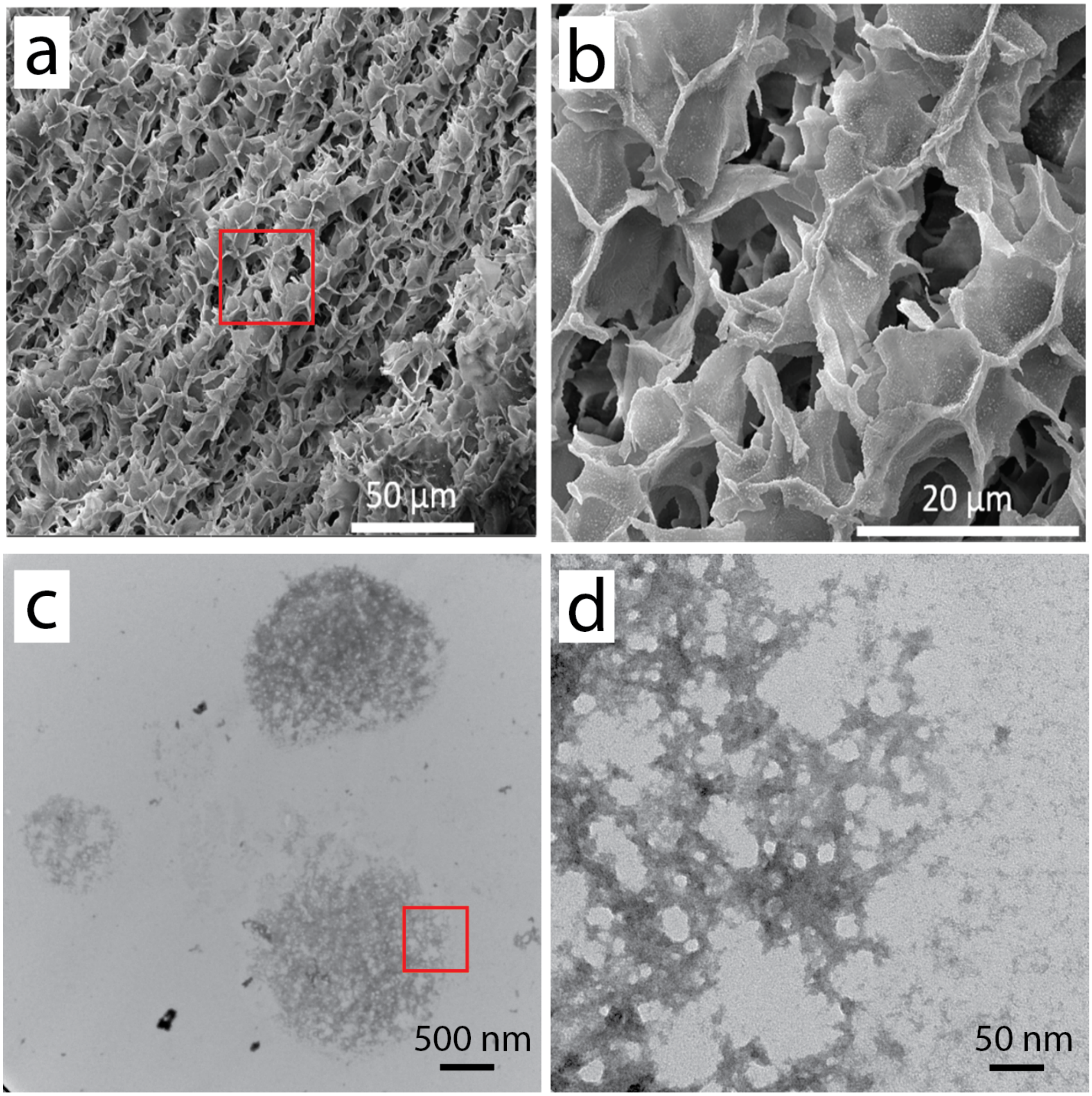

Figure S7. Scanning electron microscope (SEM) analyses (a, b) and transmission electron microscope (TEM) analyses (c, d) of poly($Lys_{80}Tyr_{20}$) hydrogels.

Small angel x-ray scattering analyses:

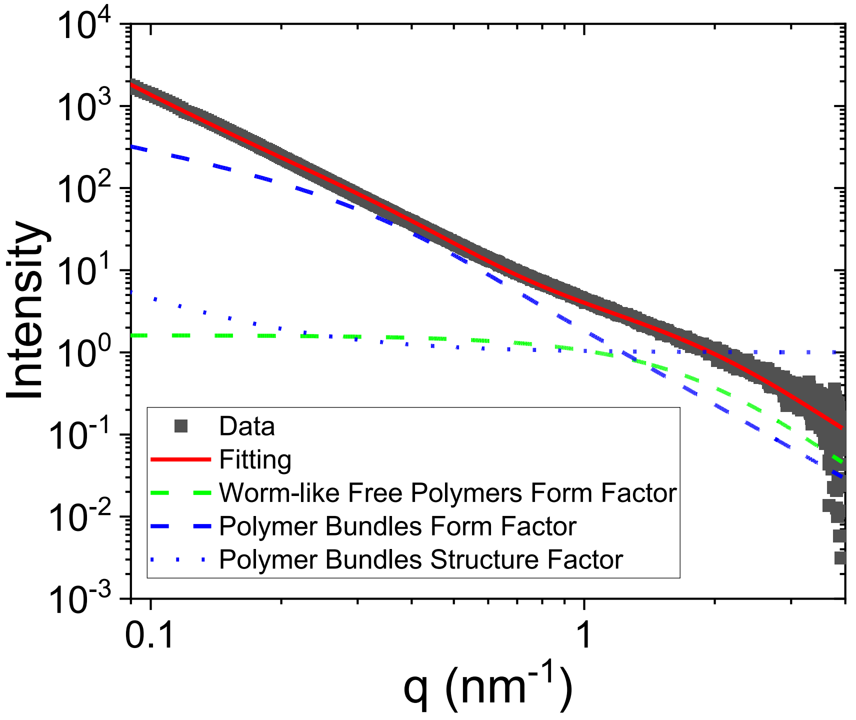

Figure S8. Fitting of scattering profiles of 3.8 mM poly($Lys_{80}Tyr_{20}$) 0.1 M PBS buffer solution after 24 hours. Individual components of the fitting are also displayed in different colour and patterns.

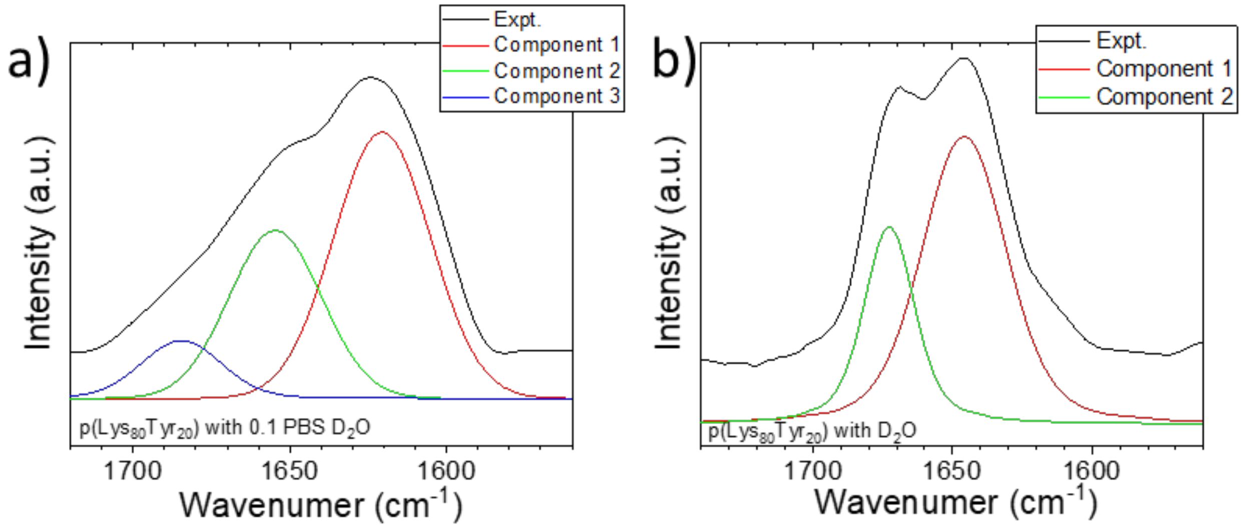

Figure S9. Relevant IR spectral deconvolution for Figure 2a

References

[1] J. Huang, C. L. Hastings, G. P. Duffy, H. M. Kelly, J. Raeburn, D. J. Adams, A. Heise, *Biomacromolecules* **2013**, *14*, 200-206.

[2] S. B. Hanay, J. O'Dwyer, S. D. Kimmins, F. C. S. de Oliveira, M. G. Haugh, F. J. O'Brien, S. A. Cryan, A. Heise, *Acs Macro Lett* **2018**, *7*, 944-949.

[3] D. Orthaber, A. Bergmann, O. Glatter, *J Appl Crystallogr* **2000**, *33*, 218-225.

[4] I. Bressler, J. Kohlbrecher, A. F. Thunemann, *J Appl Crystallogr* **2015**, *48*, 1587-1598.

[5] A. L. Kholodenko, *Macromolecules* **1993**, *26*, 4179-4183.
